# Improving the interoperability of biomedical ontologies with compound alignments

**DOI:** 10.1186/s13326-017-0171-8

**Published:** 2018-01-09

**Authors:** Daniela Oliveira, Catia Pesquita

**Affiliations:** 1 Insight Centre for Data Analytics, NUI Galway, Galway Business Park, Dangan, Galway, H91 AEX4 Ireland; 2 LaSIGE, Faculdade de Ciências, Universidade de Lisboa, Lisboa, 1749-016 Portugal

**Keywords:** Biomedical ontologies, Ontology alignment, Algorithms

## Abstract

**Background:**

Ontologies are commonly used to annotate and help process life sciences data. Although their original goal is to facilitate integration and interoperability among heterogeneous data sources, when these sources are annotated with distinct ontologies, bridging this gap can be challenging. In the last decade, ontology matching systems have been evolving and are now capable of producing high-quality mappings for life sciences ontologies, usually limited to the equivalence between two ontologies. However, life sciences research is becoming increasingly transdisciplinary and integrative, fostering the need to develop matching strategies that are able to handle multiple ontologies and more complex relations between their concepts.

**Results:**

We have developed ontology matching algorithms that are able to find compound mappings between multiple biomedical ontologies, in the form of ternary mappings, finding for instance that “aortic valve stenosis”(HP:0001650) is equivalent to the intersection between “aortic valve”(FMA:7236) and “constricted” (PATO:0001847). The algorithms take advantage of search space filtering based on partial mappings between ontology pairs, to be able to handle the increased computational demands. The evaluation of the algorithms has shown that they are able to produce meaningful results, with precision in the range of 60-92% for new mappings. The algorithms were also applied to the potential extension of logical definitions of the OBO and the matching of several plant-related ontologies.

**Conclusions:**

This work is a first step towards finding more complex relations between multiple ontologies. The evaluation shows that the results produced are significant and that the algorithms could satisfy specific integration needs.

## Background

Life sciences research is becoming increasingly integrative, with research areas such as Systems Biology and Translational Medicine bridging distinct domains to provide novel insights. The need for data integration across domains coupled with the massive amounts of data being produced both by biological and clinical domains poses new challenges. A common strategy to deal with this data deluge involves linking the information to ontologies, making it easier to search through databases and to develop algorithms to process information. Ontologies have been remarkably successful in the life sciences, especially in the biomedical domain, where the Gene Ontology [1] is the most notable success case. BioPortal^1^, a portal for life sciences’ ontologies, lists over 400 ontologies dedicated to diverse domains ranging from molecules to phenotypes.

However, when data is annotated with different ontologies, to allow data interoperability the ontologies themselves need to become interoperable. This can be achieved through a process called ontology matching [2], whereby meaningful links are established between semantically related concepts. The matching of biomedical ontologies poses specific computational challenges due to their large size and vocabulary complexity [3], and also by their increasing semantic richness in the form of new kinds of relations between classes and complex axioms. These open challenges have attracted the interest of the community and spurred the inclusion of specific tracks dedicated to biomedical ontologies in the Ontology Alignment Evaluation Initiative [4].

Currently, ontology matching techniques and systems are mostly devoted to finding links between two equivalent entities from two distinct ontologies, but when data crosses domains, the need arises for matching techniques that go beyond this and allow linking more than two ontologies through more complex relations. Compound ontology matching [5] allows the matching of several ontologies with distinct but related domains through the establishment of compound mappings that involve several entities. A specific case is the ternary compound mapping whereby two classes are related to form a class expression that is then mapped to a third class. For instance, the class HP:0000337 labelled “broad forehead” is equivalent to an axiom obtained by relating the classes PATO:0000600 (“increased width”) and FMA:63864 (“forehead”) via an intersection. Such mappings allow a fuller semantic integration of multidimensional semantic spaces, supporting more complex data analysis and knowledge discovery tasks.

In this paper, we present a set of new algorithms which are able to create ternary compound alignments for large biomedical ontologies. The algorithms were evaluated against reference ontology alignments and applied to potentially extend ontology logical definitions and to match plant ontologies.

### Related work

Ontology matching can be defined as a function *f* that returns an alignment between the classes of a pair of ontologies *O* and *O*^′^ [2]. An alignment consists of a set of correspondences (mappings) between semantically related entities of different ontologies. This process can be extended by using other parameters and resources, e.g., weights, thresholds, and even external knowledge. Most ontology matching systems usually include three main types of components: (1) loading and pre-processing, where ontology files are loaded and other procedures are employed such as normalization of labels; (2) matching, where pairs of mapped ontology entities are given a score reflecting their closeness; (3) refinement, where the list of mappings is filtered to adhere to quality, cardinality and consistency requirements among others. Typically, ontology matching corresponds to binary mappings between classes, properties or instances. However, more complex kinds of ontology matching that extend the definition have been proposed.

One of the first steps in this direction was the definition of complex ontology matching, which is commonly described as a correspondence between two classes from two different ontologies, where one of them is a complex concept or property description. It involves only two ontologies, but each mapping relates to more than two entities in those ontologies. An example of a complex mapping could be the alignment of the concept “AcceptedPaper” in one ontology, to the entity “Paper” in a second ontology, which has the associated property “Accepted” [6]. Ritze et al. [7] developed a pattern-based approach to finding these mappings, where they present correspondence patterns and define matching conditions for each of them.

The CGLUE [8] and the iMAP systems [9] were both developed to find complex matches. CGLUE applies a rule learning process and iMAP uses several searchers, each considering a meaningful subset of the space, to find complex mappings. They both apply a beam search to control the search through the space of candidate matches, given its large size.

Partial matching has also been investigated. Dhombres and Bodenreider [10] employed lexical and logical approaches to derive partial mappings for the HP ontology and SNOMED CT.

A related, but more complex approach, is compound matching [5] which is the process of identifying “compound mappings”, i.e. matches between class or property expressions involving more than two ontologies. This means that a ternary compound mapping is a tuple <X,Y,Z,R,M>, where X, Y and Z are classes from three distinct ontologies, R is a relationship established between Y and Z to generate a class expression that is mapped to X via a mapping relation M. The ontology to which X belongs is considered to be the source ontology, and the ontologies that define Y and Z are considered as the target ontologies. In this particular case, the relation R is always an intersection (regardless of any qualifier) and the mapping M an equivalence. The concept of compound alignment is defined as a set of mappings between classes from a source ontology *O*_*s*_ and class expressions obtained by combining two other classes, each belonging to a different target ontology *O*_*t*1_ and *O*_*t*2_.

To the best of our knowledge, there are currently no ontology matching systems capable of generating such mappings. However, a preliminary approach was tested by [5] that first matched the source ontology to each of the target ontologies individually, using an anchor-based word matching algorithm, and then matched all pairs of target classes that map individually to the same source class. Despite the reduced search space, they could not test their algorithm in larger sets of ontologies and evaluated only the MP-PATO-CL and MP-PATO-NBO alignments, obtaining recall values of 30 and 11% respectively, but precision values below 1%. This work was the starting point for the development of our novel approach (with preliminary results presented in [11]).

## Methods

The design, development and implementation of compound matching algorithms involved three stages: (i) an exploratory stage, which consisted in a pattern analysis of a representative set of biomedical ontologies to devise strategies and explore the challenges of the development of compound matching algorithms; (ii) the adaptation and extension of existing classical matching algorithms to compound ontology matching, which were (iii) implemented in a state-of-the-art ontology matching system.

### Pattern analysis

The first stage of this work had an exploratory nature and aimed to understand the mappings between source and target ontologies and to seek new strategies to apply to ternary compound matching. We used the ontologies for which we were able to create a reference alignment for compound matching from logical definitions of OBO ontologies (see “Reference alignments” section).

Table 1 presents these biomedical ontologies with the number of different classes and names (labels and synonyms) that each one had at the time of the download.

**Table 1:** Biomedical ontologies downloaded from the OBO Foundry in May 2015 (http://obo.sourceforge.net)

Ontology	Acronym	Classes	Names	Reference
Cell type	CL	4775	4375	[29]
Foundational model of anatomy	FMA	78977	126190	[30]
Gene ontology (biological process domain)	GO	43048	276577	[1]
Human phenotype	HP	28621	18431	[31]
Mammalian phenotype	MP	28643	29592	[32]
Neuro behaviour ontology	NBO	116710	1168	[33]
Phenotypic quality	PATO	2497	3378	[34]
Uber anatomy ontology	UBERON	18322	50713	[35]
*Caenorhabditis elegans* phenotype	WBP	2290	2739	[36]

Using a source ontology and a single target ontology as input, several binary alignments were created by applying AML’s *Word Matcher* and *String Matcher* (see the “AgreementMakerLight” section). The mappings of those alignments were manually analysed to uncover the following patterns: (1) “addition”, where the source or target class label had one or more extra words; (2) “variation”, which had labels with the same number of words, but one word did not match; (3) “combination” with mappings that combined the previous patterns; and, (4) “full match”, which had terms that match completely, but can sometimes have words in a different order. The reference alignments were also split into pairs to form binary alignments and a manual search for the previously defined patterns was performed. This search led to the discovery of a new pattern which is the occurrence of synonyms between the two classes that are being matched. Table 2 shows one example mapping for each of the situations described.

**Table 2:** Examples of the patterns found in a manual analysis of binary alignments

Pattern	Source URI and label	Target URI and label
Addition	WBP:0001911 axon regeneration *defective*	GO:0031103 axon regeneration
Variation	MP:0002269 musc*ular* atrophy	GO:0014889 musc*le* atrophy
Combination	MP:0013527 *absent* conjunctiva goblet cell*s*	CL:2000084 conjunctiva goblet cell
Full match	MP:0002119 dipsosis	NBO:0000541 dipsosis
Synonym	HP:0010108 aplasia of the *hallux*	FMA:25047 big toe
None	MP:0002229 neurodegeneration	GO:0070657 neuromast regeneration

The analysis of all the alignments led to the conclusion that the majority of the mappings fit in at least one of the pattern categories. Most, however, are a combination of the first two patterns, with the “addition” pattern being the more prevalent one (see Table 3).

**Table 3:** Distributions of mappings fitting lexical patterns 1 or 2

Matcher	Ontology	Addition	Variation	Size
String Matcher	MP-CL	26	7	34
	MP-GO	287	210	501
	MP-NBO	354	205	594
	MP-UBERON	58	11	71
	WBP-GO	182	137	322
	HP-FMA	272	23	304
	MP-PATO	18	1	29
	WBP-PATO	28	2	41
	HP-PATO	12	1	25
Word Matcher	MP-CL	4	1	5
	MP-GO	32	25	65
	MP-NBO	118	44	219
	MP-UBERON	42	5	50
	WBP-GO	183	33	219
	HP-FMA	158	44	252
	MP-PATO	33	21	59
	WBP-PATO	19	1	25
	HP-PATO	6	0	12
Reference	MP-CL	439	12	474
	MP-GO	805	83	944
	MP-NBO	177	24	219
	MP-UBERON	1693	126	1999
	WBP-GO	256	39	325
	HP-FMA	1691	66	1893
	MP-PATO	3096	35	3636
	HP-PATO	1710	8	1893
	WBP-PATO	302	4	325
	Total	12001	1168	14535

The mappings that were classified with the addition pattern are mostly partial mappings, i.e., only some words matched between the labels of the classes mapped. Dhombres and Bodenreider [10] worked on a method to identify partial lexical matches between HP and SNOMED CT. The authors used existing matching techniques and extended them to find partial mappings. Their approach identified 7358 partial lexical matches and 82% of them had an inferred logical mapping. Comparing with the 14,535 mappings analysed approximately 82% fit the addition pattern and can be considered a partial match.

These findings served as the conceptual foundation for the development of the compound matching algorithms. For instance, the prevalence of the“addition” pattern indicated that a bag-of-words approach could be an efficient solution. The existence of mapped classes with different word order, however, can change the meaning of a concept in a class and this situation would be overlooked by the bag-of-words approach. This approach would also not directly handle the synonym pattern. Finally, the variation pattern led to the use of a popular word stemmer, the Snowball stemmer^2^, which was applied to the words in each label of all the classes.

### Algorithm implementation

The Compound Matching algorithm has three main steps:

*Step 1 - First-pass recall selection.*

The algorithm performs a pairwise mapping of the labels of the source ontology with the labels of the target ontology to match first (target 1). Each word is weighted by its Evidence Content (EC) [12]. The EC is the inverse logarithm of the frequency of a word and reflects the usage of that word within the ontology. The similarity is then calculated by finding the ratio between the sum of the EC of the words shared by the source label (*l*_*s*_) and the target 1 label (*l*_*t*1_), and the sum of the EC of the words in *l*_*t*1_.

1 $$ sim_{1}\left(l_{s},l_{t1}\right) = \frac{\sum EC\left(word \in \left(l_{s} \cap l_{t1}\right)\right)}{\sum EC\left(word \in l_{t1}\right)}  $$

*Step 2 - Search space reduction.*

The algorithm filters out all mappings with similarity below a given threshold and removes all the source classes which were not mapped to any target 1 classes. It also reduces the number of words of the source labels by removing from the mapped classes all the words that had a match with a word from a target 1 class. Taking the example of “Aortic valve stenosis” (HP:0001650), after matching HP with FMA, which would capture the mapping for “aortic valve” (FMA:7236), HP’s class label would be reduced to “stenosis”.

*Step 3 - Longest match precision selection.*

For each of the remaining mappings, the algorithm performs a pairwise mapping of the reduced source labels against the labels of the last target (target 2). In this step, however, the denominator corresponds to the sum of EC of the words in the longer label, to ensure a complete match.

2 $$ sim_{2}\left(l_{s},l_{t2}\right) = \frac{\sum EC\left(word \in \left(l_{s*} \cap l_{t2}\right)\right)}{\sum EC\left(word \in longest \left(l_{s},l_{t2}\right)\right)}  $$

The final similarity between the matched labels is computed as the average between the similarities computed in steps 1 and 2. Mappings with an average below the second threshold are filtered out.

The resulting alignment is a list of all mappings above the selected threshold, without any consideration for cardinality. To ensure proper cardinality, refinement (or selection) strategies need to be employed. The reference alignments have a cardinality of 1, meaning that for each source class there is a single compound mapping. However, given the potential for conflicts, it was also desirable to investigate the option of allowing two mappings for the same source class. To this end, both a top-one and top-two ranked selectors were implemented.

Both are greedy algorithms that select mappings based on their similarity. They start by sorting the mappings in the compound alignment in descending order of their similarity values. When there are competing mappings with equal similarity values, the top-one selector chooses a single mapping taking the one that was sorted as first, whereas the top-two selector, chooses the two first sorted mappings.

### AgreementMakerLight

The AgreementMakerLight (AML) ontology matching system [13] focuses on the efficient matching of very large ontologies and is one of the most successful systems for aligning ontologies [14]. AML has three main modules: (1) ontology loading, (2) ontology matching and (3) alignment selection and repair. When an ontology is loaded into AML, a *Lexicon* is built with all class labels and synonyms. AML has several matchers that explore lexical and structural information. The selection and repair module ensures that the final alignment has the desired cardinality and removes mappings causing logical inconsistencies.

In this work, we adapted the loading module to handle three ontologies. The implementation of our matching algorithm takes advantage of the data structures AML builds for its *Word Matcher*. The *Word Matcher* uses a bag-of-words approach and creates a new *Lexicon* with every word frequency and EC. The similarity between classes of different ontologies is then based on a weighted Jaccard index. We also made use of AML’s selection strategies, which were adapted to work over compound mappings.

### Evaluation

The compound alignments were evaluated with classification metrics automatically against reference alignments and also manually, to better understand the results and point towards possible improvements.

#### Reference alignments

The technique for the construction of the compound reference alignments used in the evaluation originated from the work of [5], where ternary compound mappings were derived from logical definitions of OBO ontologies to be used as a gold-standard.

Logical definitions are applied to classes and use genus-differentia constructs of the form “X is a G that D”, where X is the defined class, G is the genus and D the differentia. The genus is a more general class than X and D discriminates instances of X from other instances of G [15]. The following text shows an example of a logical definition:



OBO ontologies with over 100 logical definitions that had a class expression intersected by two classes from two other ontologies were selected (see the example above). Following these rules, we created six reference alignments, which determined the sets of biomedical ontologies used throughout this work.

#### Precision, recall and F-measure

The automatic evaluation of the algorithms was performed based on the classification metrics precision, recall and F-measure, which are defined in this context as follows.

$$ Pr(A) = \frac{|\left\{ m | m \in A \wedge m \in R \right\}|}{|A|} $$

3 $$ Rec(A) = \frac{|\left\{ m | m \in A \wedge m \in R \right\}|}{|R|}  $$

4 $$\begin{array}{*{20}l} F\text{-}measure(A) = 2 \cdot \frac{Pr(A) \cdot Rec(A)}{Pr(A) + Rec(A)} \end{array} $$

where *A* is an alignment resulting from the algorithms developed, *Pr*(*A*) and *Rec*(*A*) are the precision and recall, respectively, of the alignment. *m* is a mapping in an alignment. *R* is the reference alignment to which *A* is compared.

#### Thresholds

The thresholds used in the evaluation process were defined through a series of tests aimed to find consistent values across all six sets of ontologies, which returned the best metrics.

The evaluation process involved testing the algorithms with different thresholds for the first and third algorithm steps and checking which were the two optimal values to use throughout the evaluation process. These values had to return good precision or recall but also needed to have a reasonable runtime with a considerable amount of mappings found^3^.

The first-pass recall selection needs to return a high recall so that the search space is not too narrowed for the subsequent steps of the algorithm while still providing good filtering of irrelevant mappings. For this to happen the first threshold (*T*_1_) needs to be low to return a high recall, at expense of the precision. To determine the best *T*_1_ the compound reference were reduced to binary references by removing the second target from each class. These binary references were compared with the mappings resulting from the first-pass recall selection in terms of recall, total number of mappings found and runtime.

**Fig. 1:**
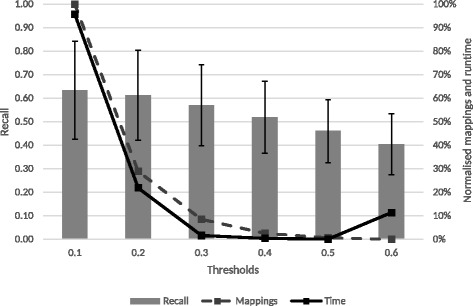
First-pass recall selection for the first threshold. The left axis shows the values for recall and the right axis shows the normalised averages for the runtime and number of mappings across all sets of ontologies

Figure 1 presents the results for the first-pass recall selection experiments with *T*_1_ equal to 0.1, 0.2, 0.3, 0.4, 0.5 and 0.6. This range was selected since thresholds above 0.6 are commonly used thresholds for full equivalence binary mappings. Since all ontology sets have different sizes, to compare the mappings and runtime, their values were normalised to obtain result from 0 to 1. They were then averaged over all sets for each threshold to obtain the overall variation of mappings and time.

Figure 1 shows that between the thresholds 0.1 and 0.3 there is a steep decline in both time and number of mappings. This decline, for example for the HP-FMA-PATO set, reflects a reduction from 73 h to 51 min and for the MP-UBERON-PATO set from 2.5 h to 44 s. Therefore, both 0.1 and 0.2 were excluded for the *T*_2_ tests. The 0.6 threshold was also not considered for the tests for *T*_2_ since it is the threshold with less mappings, lowest recall and it does not improve the runtime.

The *T*_2_ tests used the longest match precision selection step to find the best combination of first and second thresholds. Using the set of *T*_1_ [0.3, 0.4, 0.5] in combination with the set of *T*_2_ [0.7, 0.8, 0.9], the final thresholds selected to run the evaluation of the algorithm were the ones that returned the best precision, while still considering recall and runtime.

**Fig. 2:**
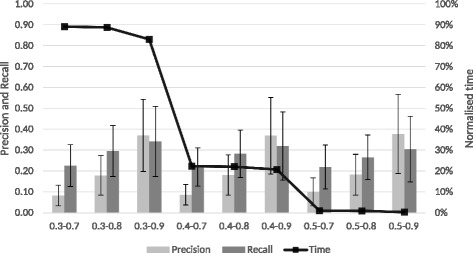
Longest match precision selection for the second threshold. Longest match precision selection for the second threshold. The left axis indicates the values for precision and recall and the right axis shows the normalised averages across all ontology sets for the runtime

Figure 2 shows the results for the longest match precision selection with different combinations of thresholds. From the set of *T*_2_ tested, 0.9 consistently achieves the higher precision and recall. In combination with the first threshold, 0.3 achieves slightly higher recall than 0.4 but has a runtime around 4 times higher. This increased runtime translates, for example, for the set HP-FMA-PATO in a reduction from 2.5 to 52 min or for the set MP-UBERON-PATO the runtime decreases from 57 min to 19 min. The 0.5-0.9 thresholds achieve even lower recall than previous combinations even though the time difference is still significant. For example, the HP-FMA-PATO set runs in 2 min, while the MP-UBERON-PATO finishes in 8 min.

Considering these experiments, for the remaining evaluation of the algorithms, the thresholds used were *T*_1_=0.4 and *T*_2_=0.9. The first threshold was chosen for its high recall and the second for its high precision. This combination achieves a high precision, while maintaining a good recall and both thresholds achieve a reasonable runtime.

## Results

### Automated evaluation

The first evaluation consisted in the comparison of the alignments obtained through the algorithms with the compound reference alignments by analysing precision, recall and F-measure.

Table 4 contains the precision, recall and F-measure values of the compound alignments using the top-one ranked selector. The results show that the precision is consistently higher than the recall, with the highest F-measure being 61.8% for MP-GO-PATO and the lowest 10.9% for HP-FMA-PATO. However, the algorithms still have a low performance, with only three sets achieving a precision over 50% and only one with a recall over this mark.

**Table 4:** Evaluation results from the comparison with the automatically generated reference alignments with the Top-one Ranked Selector. The “Ref.” column indicates the number of mappings present in the the reference alignments

Ontology sets	Precision	Recall	F-measure	Ref.
MP-CL-PATO	24.5%	24.3%	24.4%	474
MP-GO-PATO	62.9%	60.7%	61.8%	944
MP-NBO-PATO	50.0%	39.7%	44.3%	219
MP-UBERON-PATO	55.2%	46.8%	50.7%	1999
WBP-GO-PATO	11.7%	10.2%	10.9%	325
HP-FMA-PATO	27.3%	20.3%	23.3%	1893

The top-two ranked selector was developed and applied after the manual analysis showed that most of the correct mappings could be found, if not in the first position, immediately on the second. Table 5 shows that the top-two selector returns higher recall values for all ontology sets, sometimes at the expense of the precision. The MP-CL-PATO was also the only set which obtained a significantly higher precision with the top-two selector than the top-one selector. Four of the six sets of ontologies obtained a higher F-measure due to the marked increase in the recall values.

**Table 5:** Evaluation results from the comparison with the automatically generated reference alignments with the top-two ranked selector

Ontology sets	Precision	Recall	F-measure	Ref.
MP-CL-PATO	34.9%	53.0%	42.0%	474
MP-GO-PATO	41.5%	61.5%	49.6%	944
MP-NBO-PATO	42.7%	41.1%	41.9%	219
MP-UBERON-PATO	52.8%	51.4%	52.1%	1999
WBP-GO-PATO	11.6%	13.5%	12.5%	325
HP-FMA-PATO	24.0%	22.9%	23.4%	1893

Both the increase in recall and decrease in precision can be explained by the presence of two mappings for some source classes instead of one. For example, both the mapping “retinal ganglion cell degeneration” (MP:0008067) with “retinal ganglion cell” (CL:0000740) and “degeneration” (PATO:0002037) and the mapping “retinal ganglion cell degeneration” (MP:0008067) with “retinal ganglion cell” (CL:0000740) and “degenerate” (PATO:0000639) are present in the alignment with the top-two selector. When using the top-one selector, however, only one of these mappings will be present in the alignment. Since both mappings have the same similarity, the one chosen for the final alignment will be randomly selected and it is not necessarily the one featured in the reference alignment. Thus the presence of both mappings reduces the precision, because one of them is always wrong, but increases the recall, since the alignment covers more mappings from the reference.

The MP-CL-PATO benefits the most from the top-two selector because it is the set which contains more of these competing mappings. The prevalence of similar mappings points to the possibility of the existence of disagreement between reality models in the source and target ontologies, i.e., sister classes in one ontology might be considered synonyms in another. For instance, “present in fewer numbers in organism” (PATO:0001997) is a sister class of “has fewer parts of type” (PATO:0002001) and, despite having different definitions, both have “decreased number” as an exact synonym. Thus, when matching this ontology to others, these two classes can have the same meaning and lead to the presence of competing mappings. These mappings create the need for two-to-one alignments, and a selection algorithm such as the top-two selector will thrive in these kinds of alignments since it can cover more possibilities than the top one ranked selector.

### Manual evaluation

The manual evaluation focused on the mappings that were not found in the reference alignment. In a first step, mappings generated by the top-one Ranked selector were assigned into one of three categories: “correct mappings, “conflicting mappings” and “missing mappings”. Correct mappings match the same two target classes to the same source. Conflicting mappings are the ones whose source class is contained in the reference alignment, whereas missing mappings are those for which the source class is not present in the reference alignment.

Table 6 shows the number of mappings that fit in each one of these categories. Three of the sets (MP-GO-PATO, MP-NBO-PATO and MP-UBERON-PATO) have the most mappings matching exactly the reference, while the remaining three sets either have a majority of mappings missing from the reference or a majority of conflicts.

**Table 6:** Comparison of the compound alignments and the compound references

Ontologies	Correct	Missing	Conflict
MP-CL-PATO	132	158	158
MP-GO-PATO	556	204	79
MP-NBO-PATO	84	35	50
MP-UBERON-PATO	831	390	192
WBP-GO-PATO	31	105	140
HP-FMA-PATO	482	611	196

A random subset of 40 mappings from each matching task was selected with 25 being conflicting mappings and 15 missing mappings. These were analysed by two independent human annotators, with 5 year graduate studies in life sciences, who categorised the conflicting mappings into three mutually exclusive classes (“more correct in reference”, “more correct in alignment”, or “equally correct in both”), and the missing mappings into two mutually exclusive classes (“correct”,“incorrect”). The annotators are experienced users of biomedical ontologies and were asked to check class definitions and ancestry when making their decision. The agreement between the annotators was calculated as the proportion of mappings classified into the same category by both annotators. The results of this analysis are presented in Table 7.

**Table 7:** Manual evaluation of mapping subsets

Ontologies	Conflicts				Missing		
	Reference	Alignment	Both	Agreement	Correct	Incorrect	Agreement
MP-CL-PATO	0.0%	3%	97%	87%	62%	38%	88%
MP-GO-PATO	60%	30%	10%	41%	60%	40%	76%
MP-NBO-PATO	47%	40%	13%	100%	78%	22%	72%
MP-UBERON-PATO	20%	40%	40%	67%	84%	16%	84%
WBP-GO-PATO	7%	20%	73%	80%	74%	26%	64%
HP-FMA-PATO	3%	44%	53%	75%	92%	8%	100%

Table 7 shows that, with relatively high agreement, most of the conflicts are considered correct in both the alignment and the reference or more correct in the alignment. For our analysis, we will consider these mappings as true positives. The exception is the MP-GO-PATO set were the sum of the potentially correct mappings is still lower than the mappings considered correct in the reference. This is also the only set where the agreement between the annotators is below 50%.

The high number of conflicts potentially correct in both the alignment and the reference occurs, for example, in the MP-CL-PATO, with mappings which involve a reference to an increased/decreased number of cells. The algorithms always match those cases to “decreased amount” (PATO:0001997)/“increased amount” (PATO:0000470) instead of “has fewer parts of type”(PATO:0002001)/“has extra parts of” (PATO:0002002), which are the classes present in the reference alignment. The conflicts potentially more correct in the alignment occur, for example, in the set MP-UBERON-PATO with the mapping “flattened snout” (MP:0000447) with “snout” (UBERON:0006333) and “flattened” (PATO:0002254), which could be considered more accurate than the mapping present in the reference alignment which is “flattened snout” (MP:0000447) with “midface” (UBERON:0004089) and “flattened” (PATO:0002254).

For the mappings missing from the reference, with high agreement, most of them are considered correct. An example of a mapping considered correct from the MP-UBERON-PATO set is “absent thoracic vertebrae” (MP:0004655) with “thoracic vertebra” (UBERON:0002347) and “lacks all parts of type” (PATO:0002000).

## Discussion

One challenge of computing compound alignments is the memory requirements involved in the process. If matching two large biomedical ontologies is already challenging for many ontology matching systems, handling three ontologies in a compound alignment scenario is even more demanding. The algorithms here presented decrease the search-space by using a three-step matching approach, which both reduces the time and memory requirements.

The algorithms were tested automatically and manually. While the automatic results against the reference alignments underperformed, with lower than expected statistics, the manual evaluation showed that the algorithms were in most cases returning a majority of correct mappings. This indicated, on one hand, that the reference alignments are incomplete, and on the other hand, that the algorithms are failing to capture a considerable portion of reference mappings. As ontologies are constantly evolving, evaluations made against more complete versions can potentially provide better results. Moreover, this evaluation also led us to find several mappings which conflicted with the mappings present in the reference alignment. Using the top-two ranked selector allowed some of the alignments to overcome the issue by featuring both the conflict and the non-conflicting mappings in the alignments at the expense of the precision. This selection strategy can be used in a user interaction scenario, where the user then decides between the two conflicting mappings.

Despite a clear variability in performance between ontology sets, the manual evaluation showed that in 4 out of 6 matched sets the precision of conflicting mappings is greater than 80% and for missing mappings it is 74%.

Notwithstanding the overall good performance of the proposed algorithms, there are still some open challenges. Theoretically, the main compound matching algorithm is symmetric, i.e., the target order does not matter. However, we empirically found that the algorithm performs better if the ontologies are aligned in a specific order. In this case, we always matched PATO last since we consistently obtained better results with this specific order. In the future, it would be desirable to automate the selection order by evaluating the coverage of each of the matching orders.

Currently, the user needs to possess specific previous knowledge of the ontologies to be able to perform the alignment, i.e., the user needs to know which two ontologies are able to form a set of terms which is equivalent to the label of the source ontology. One solution to this challenge could be to use several ontologies as input to automatically determine the ontologies which could form potential compound mappings. AML currently uses a similar strategy to determine which ontologies can be used as background knowledge in a binary alignment setting [16], which can in principle be adapted to select the appropriate ontologies for compound matching.

Exploring external knowledge with existing techniques proved to be too computationally demanding. However, given the fact that some mappings need external knowledge to be identified, there is still a need to adapt these strategies to a ternary compound matching setting.

In addition, the proposed methodology does not explicitly handle the possibility that there are binary equivalence mappings between ontology pairs. This could eventually occur if a concept in the source ontology also occurs in one of the targets. However, from a practical point of view and considering that for biomedical ontologies a high proportion of equivalence mappings is found through string and lexical approaches, the intermediate step of the algorithm prevents these mappings from occurring in the alignment. This is ensured by removing words already matched to the first target, and considering that it is likely that a high proportion of equivalence mappings would involve all of the words in a class label, this would result in the mapping between source and first target not proceeding to the next step of the algorithm. Nevertheless, the proposed methods could be combined with a pre-processing step where binary matching algorithms would be applied to identify redundancies to remove.

### Applications

#### Logical definitions

One notable effort in increasing the interoperability of ontologies has been the creation of logical definitions. Almost all classes in a biomedical ontology have a textual definition, which can be interpreted by a human user but cannot be easily accessed by a computer without sophisticated natural language processing. Therefore, efforts have been made to transform these definitions into a computable form as a set of logical definitions. However, creating, implementing and maintaining these computable definitions can be difficult, as it requires a lot of manual labour. The creation of the logical definitions was partially automated with Obol [17], a tool that applies a set of fairly complex ontology-specific grammar rules to generate proposed logical definitions from pre-existing classes, which are then vetted by experts. Using this approach the authors managed to map 73% of MP classes, 19% of WBP, 20% of HP and 42% of the Plant Trait Ontology, using PATO. In a later work, Obol was also applied to the creation of logical definitions for the GO to improve its integration with other OBO ontologies [15]. These logical definitions were then used as an input for the creation of the Cellular Phenotype Ontology [18], which is an ontology that describes cells and their associated processes and, thus, provides uniform definitions to annotate cellular phenotypes.

The significant percentage of new mappings and conflicts revealed by the manual evaluation (see Table 7) led us to investigate how the algorithms could impact the current state of the OBO logical definitions since the reference alignments were based on them. Our proposal is that ternary compound matching could be used to identify candidates for logical definitions, which could then be refined through the application of reasoning and expert validation. Table 8 compares the number of new and conflicting mappings produced for each of the three ontologies in relation to the total number of OBO classes represented in the logical definitions.

**Table 8:** Candidate logical definitions

Ontology	New mappings	Conflicts	OBO classes
MP	335	442	7694
WBP	72	140	957
HP	498	169	14059

These mappings differ from the ones in Table 7, since here the “New” mappings are classes from the source ontology that are not present in the respective logical definitions, which puts some of the mappings previously classified as “New” in the conflict column. These mappings correspond to non-ternary logical definitions that were excluded from the reference alignments.

Table 8 shows that there are 335 candidate logical definitions to be considered for the MP logical definitions. The WBP could have 72 new candidate logical definitions and the HP ontology 498. This represents more than 900 new candidate logical definitions for classes that had none.

However, the algorithms also produced more than 750 mappings that are in conflict with the logical definitions. Over 400 of these correspond to non-ternary logical definitions. For instance, the logical definitions of the MP ontology contains the following:



This logical definition is present in the alignment as “absent cochlear outer hair cells” (MP:0004403) with “outer hair cell” (CL:0000601) and “lacks all parts of type” (PATO:0002000). This mapping is not erroneous since “cochlear outer hair cells” is an exact synonym of this label. However, because a logical definition for this source class existed, this mapping was added to the number of conflicts. Cases such as these showcase the possibility of producing more than one correct logical definition for each class. Besides these non-ternary conflicts, the ternary conflicts still account for more than 300 possible new logical definitions. Therefore, an expert analysis of the conflict mappings can potentially reveal novel logical definitions that can further improve the ontologies.

Both the consideration of new mappings and the analysis of the conflict mappings could lead to the improvement of logical definitions. This method could go beyond the currently employed methods since it can find new potential logical definitions, and propose improved alternatives to some pre-existing ones.

One relevant consideration is that the candidate logical definitions once integrated into the ontologies can cause logical inconsistencies. Logical inconsistencies due to the integration of two ontologies with different modelling views is a well known challenge in the biomedical ontology matching area [19, 20]. There are several approaches to detect and repair these issues in binary ontology matching [21, 22]. Applying these types of approaches could further improve the quality of candidate logical definitions. However, applications of compound matching that do not require integrated reasoning over the ontologies may not benefit from enforcing logical consistency due to information loss [23].

#### Crop ontology

One of the tasks in the “Planteome” project^4^ involved aligning the Wheat Crop Ontology [24] to reference ontologies Trait Ontology (TO) [25], Plant Ontology (PO) [26] and PATO. Their initial plan was to use the standard AML matchers to complete the task. However, they needed to find more complex matches, such as “leaf length” (CO:321_0000044) with “leaf” (PO:0025034) and the PATO class “length” (PATO:0000122), since their purpose is to create formal definitions for plant traits which would allow reasoning over these ontologies. The novel proposed compound algorithms were applied to the tasks of aligning CO-PO-PATO and TO-PO-PATO. To find the optimal thresholds for this new data, we performed similar tests to the ones presented in the *Thresholds* section, but since there is no reference alignment available, the alignments were manually checked and the thresholds chosen were the ones which resulted in a reasonable amount of mappings found, with a significant number of correct results.

Table 9 shows a representative selection of the most promising pairs of thresholds with the results regarding the two sets of ontologies tested. It presents the number of mappings found and the percentage which we considered correct. For TO-PO-PATO the highest percentage of correct mappings was found using 0.1/0.9 as thresholds (96%), which could also be considered the best thresholds for CO-PO-PATO since the algorithms find more mappings (14) and keeps a relatively high percentage of correct mappings (93%).

**Table 9:** Evaluation of the plant based alignments

T1	T2	CO-PO-PATO			TO-PO-PATO		
		Found	Correct	Time	Found	Correct	Time
0.1	0.9	14	93%	20s	259	96%	149s
0.1	0.7	45	36%	20s	487	55%	169s
0.3	0.85	4	100%	6s	152	95%	15s
0.5	0.9	0	0%	5s	25	92%	7s

The few adjustments needed to make the algorithms run optimally to obtain significant results were positively unexpected since the whole project was designed to work with large biomedical ontologies. These ontologies rely mainly on narrow synonyms. Therefore, the only adjustment to the algorithm was to give a higher weight to narrow synonyms since it was too low and was severely reducing the final similarity of the alignments.

These experiments illustrate that the proposed algorithms can be generalized to other life sciences ontologies with positive results.

## Conclusion

Biomedical ontologies are crucial to support the management and analysis of life sciences data. Classical binary ontology matching techniques have been used to ensure interoperability between ontologies covering the same or closely related domains. However, broadening the concept of ontology matching is necessary to handle the complex relations in biomedical ontologies, to which we contribute with the first efficient and effective algorithms for the compound matching of three distinct ontologies. Despite the promising results, the algorithms still present some shortcomings mainly due to the computational intensiveness of the matching process which poses challenges in expanding the algorithms to take into account for instance word order or external resources. Another limitation is the definition of the relations between mapped concepts. Currently, the algorithm is using simple relations (e.g. “intersection”) to classify the relationship between the mapped concepts, but it could potentially support more complex relations such as location. Another possible direction for improvement is the development of logical repair techniques that are able to remove ternary mappings causing inconsistencies.

In the future, compound ontology matching could also be adapted to the integration of multidimensional semantic spaces [27], where it could enable the creation of more complex semantic annotations and it would allow the discovery of new and interesting associations of concepts from multiple dimensions. Compound ontology matching could also be used to help in the creation of new logical definitions with ternary intersections, as shown by the results. This application, however, would need to be enhanced by the use of ontology design patterns [28] to take into account all the constraints that are required for the development of new ontologies.

## Abbreviations

AML: AgreementMakerLight
CL: Cell ontology
CO: Wheat crop ontology
EC: Evidence content
FMA: Foundation model of anatomy ontology
GO: Gene ontology
HP: Human phenotype ontology
MP: Mammalian phenotype ontology
NBO: Neuro behavior ontology
OBO: Open biomedical ontologies
PATO: Phenotypic quality ontology
PO: Plant ontology
Ref.: Reference alignments
TO: Plant trait ontology
UBERON: Uber anatomy ontology
WBP: C.elegans phenotype ontology

## Footnotes

1
http://bioportal.bioontology.org

2
https://snowballstem.org

3 Experiments ran with an Intel^®;^ Xeon^®;^ E5606 2.13GHz with 8 cores and 94 GB of memory.

4
http://planteome.org/
